# iResNetDM: An interpretable deep learning approach for four types of DNA methylation modification prediction

**DOI:** 10.1016/j.csbj.2024.11.006

**Published:** 2024-11-13

**Authors:** Zerui Yang, Wei Shao, Yudai Matsuda, Linqi Song

**Affiliations:** a Department of Chemistry, City University of Hong Kong, Hong Kong; b City University of Hong Kong Shenzhen Research Institute; c Department of Computer Science, City University of Hong Kong, Hong Kong

**Keywords:** Deep learning, DNA modification, Interpretable analysis

## Abstract

**Motivation:**

Although several computational methods for predicting DNA methylation modifications have been developed, two main limitations persist: 1) All of the models are currently confined to binary predictors, which merely determine the presence or absence of DNA methylation modifications and thus prevent comprehensive analyses of the interrelations among varied modification types. Multi-class classification models for RNA modifications have been developed, and a comparable approach for DNA is essential. 2) Few previous studies offer adequate explanations of how models make decisions, instead relying on the extraction and visualization of attention matrices, which have identified few motifs and do not provide sufficient insights into the model decision-making process.

**Result:**

In this study, we introduce the task of DNA methylation modification prediction as a multi-class classification problem for the first time. We present iResNetDM, a deep learning model that integrates Residual Networks (ResNet) with self-attention mechanisms. To the best of our knowledge, iResNetDM is the first model capable of distinguishing between four types of DNA methylation modifications. Our model not only demonstrates good performance across various DNA methylation modifications but can also capture relationships between different types of modifications. We used the integrated gradients technique to enhance the interpretability of the iResNetDM. This method can effectively elucidate the model’s decision-making process, thus enabling the successful identification of multiple motifs. Notably, our model displays remarkable robustness, and can effectively identify unique motifs across different methylation modifications. We also compared the motifs discovered in various modifications and found that some had notable sequence similarities, suggesting that they may be subject to different types of modifications. This finding highlights the potential importance of these motifs in gene regulation.

## 1 Introduction

Epigenetic modifications, which encompass mechanisms such as histone alterations [40] and the regulation of nucleosome positioning and density [24], constitute heritable changes that do not stem from modifications in the DNA sequence itself but rather from the dynamic shifts in DNA accessibility and the architecture of chromatin. Among these, DNA methylation stands out as a critical epigenetic mechanism that is instrumental in the regulation of gene expression and the safeguarding of genomic stability. Comprehensive research on DNA methylation reveals that it is indispensable in critical biological processes, including embryonic development, X-chromosome inactivation, and genomic imprinting. Aberrations in methylation patterns are implicated in a spectrum of diseases, with cancer being a prominent example. In malignancies, abnormal hypermethylation can silence tumor suppressor genes [17], whereas hypomethylation may activate oncogenes [30].

Among these modifications, methylation at the sixth position of adenine (6-methyladenine or 6 mA) has been observed in prokaryotic and eukaryotic organisms, and contributes to the regulation of DNA replication and repair [33]. Similarly, 5-methylcytosine (5mC) [5] and its derivative 5-hydroxymethylcytosine (5hmC) [8] are well-recognized in mammalian DNA, and play significant roles in gene silencing and embryonic development. Cytosine–guanine dinucleotides are a typical example of 5mC methylation. In vertebrate genomes, around 80 % of all CpG dinucleotides contain 5mC [2]. In addition, 4-methylcytosine (4mC), although less common, has been identified in certain bacteria as part of their unique epigenetic landscapes [18]. Together, these modifications constitute a complex layer of regulatory mechanisms that are essential for the subtle regulation of genetic function and heritable phenotypic variation.

## 2 Related work

Current experimental-based techniques for identifying these modifications, such as methylated DNA immunoprecipitation (MeDIP) [27] and mass spectrometry [10], can be time- and labor-intensive. Several computational methodologies have emerged in recent decades as alternatives to traditional wet-lab techniques. Most machine learning methods are based on the support vector machine, the Markov model, or the decision tree [15], [22], [23]. In terms of deep learning, language models and the convolution neural network are widely applied to predict 4mC [35], 6 mA [41] and 5mC [7], respectively. The recent advent of the transformer model and self-attention mechanism [31] has markedly enhanced model performance in predicting DNA modifications [1], [28], [32], [36].

Despite these advances, most of these models are tailored to recognize only a single type of DNA methylation modification (DMM). A notable exception is iDNA-MS [20], which collects datasets from various databases and is the first model to apply machine learning to generically predict three DMM types, namely 4mC, 5hmC, and 6 mA, of various species. This pioneering study investigates the impact of diverse sequential features, such as the K-tuple nucleotide frequency component, nucleotide chemical properties, nucleotide frequency, and mono-nucleotide binary encoding, and assesses the efficacy of several machine learning classifiers including naïve Bayes, Bayes net, and decision tree. Building on iDNA-MS, subsequent deep learning models like iDNA-ABT, iDNA-ABF, and StableDNAm [16], [37], [42] have further honed the precision of DMM site detection across multiple species. However, these models are predominantly combinations of different binary classifiers, with each only responsible for one specific type of methylation modification of one specific organism. Thus, the training processes of different methylation modifications are totally independent, and therefore research on the relationships or interactions among these modifications is limited. In parallel, TransRNAm [6] leverages a Transformer combined with a convolutional neural network (CNN) framework to detect 12 distinct RNA modifications, with only one model containing all 12 MLP modules, each dedicated to a single modification type. This approach, while effective for RNA, highlights the absence of a similar tested methodology for DNA. Thus, a proficient multi-class DMM predictor is required to fill this gap.

iDNA-ABF [16] addresses the lack of sufficient interpretability regarding the decision-making processes of most models. In their analysis of the model’s prediction process, the authors selected three sequences from datasets, which represent 6 mA, 5mC, and 5hmC modifications, respectively, and thus essentially there is only one sequence per modification. They then visualized the attention maps and identified nucleotides with high attention scores as the regions deemed important by the model. However, this analytical approach has two major limitations. First, it cannot accurately measure the importance or contribution of individual nucleotides because k-mer aggregation has been applied, and each position in the attention matrix represents a combination of k nucleotides. Second, the sample size is too small to provide a comprehensive and adequate analysis.

In this study, we introduce iResNetDM, which to the best of our knowledge is the first deep learning model designed to predict specific types of DNA modifications rather than merely detecting the presence of modifications. iResNetDM integrates a ResNet [14] with a self-attention mechanism. The incorporation of ResNet blocks facilitates the extraction of local features and empowers the model to achieve a deeper representation of the sequences. Compared with the baseline model, iResNetDM exhibits significant enhancements across various performance metrics, achieving high accuracy across all DNA modification types and species. This demonstrates the model’s robust adaptability and efficiency in diverse biological contexts. The main contributions of our work are summarized below.

1. Current models can handle at most three DNA methylation modification types simultaneously, i.e., 4mC, 6 mA, and 5hmC [16], [37], [42]. Our model extends these by adding 5mC.
2. By leveraging the integrated gradients (IG) technique [26], we identify several motifs for the same datasets that have not been reported in other studies. This discovery provides further insights into the decision-making processes of the model.
3. Taking advantage of the multi-class framework of our model, we identify strong relationships and interactions among some of the modifications.
4. In our research, we demonstrate that ResNet, although infrequently utilized in this domain, plays a crucial role in enhancing the model’s performance, thereby underscoring its effectiveness and practicality for such tasks. This provides additional options for future studies.

## 3 Materials and method

### 3.1 Dataset selection and pre-processing

**Fig. 1:**
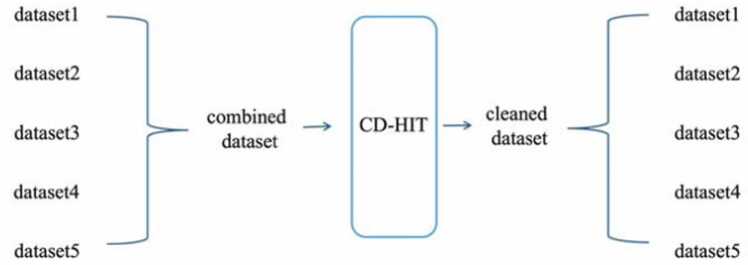
Data pre-processing procedure.

The datasets utilized in our study were derived from a source [20] that comprises 17 datasets across 12 species. We randomly selected parts of datasets that were significantly larger than others. Given that 6 mA is the sole modification occurring on adenine, 6mA-negative datasets sourced from Lv et al. [20] were also included to enrich the learning process regarding 6 mA. The 5mC dataset for *Z. mays* and *Oryza sativa* Japonica Group cv. Nipponbare (NIP) was sourced from Zhang et al. [39]. The combined datasets were input into CD-HIT [12] and those with 80 % similarity were removed (Fig. 1). To the best of our knowledge, our dataset compilation encompasses all of the major known DNA methylation modification types. The details of the datasets can be found in the Supplementary Table S1.

All samples were 41 bp in length with a central modified C or A, flanked by 20 bp regions. Additionally, akin to BERT’s training [9], a [CLS] token was prepended to each sequence before embedding to facilitate subsequent classification.

### 3.2 Model architecture

**Fig. 2:**
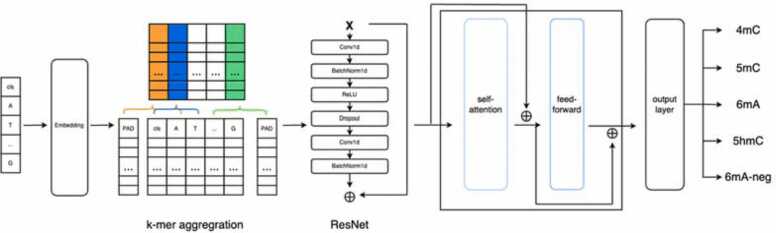
Architecture of iResNetDM. Embedded DNA sequences are initially fed into a ResNet layer, which is tasked with extracting and learning local features. Subsequently, these features are processed by a self-attention layer designed to capture global features. Finally, a fully connected layer synthesizes these insights to produce the output.

The architecture of our model (Fig. 2) comprises four core components: (1) the embedding and k-mer aggregation layer, (2) the ResNet layer, (3) the self-attention layer, and (4) the fully connected layer, which synthesizes the output. Each nucleotide in a DNA sequence was initially converted into a 256-dimensional vector. A k-mer aggregation, which uses a Conv1d layer with kernel size 3, was then utilized to enhance the sequence representation, thus allowing each nucleotide to capitalize on the neighboring information. The sequence then underwent a ResNet framework comprising five identical modules that were designed to both extract and synthesize local features, while preserving maximal original data. The output from the ResNet section was then processed through a self-attention mechanism, thus assembling a global context. Finally, the learned representation of the DNA sequence was input into a fully connected neural layer, which utilizes a softmax activation function to execute the prediction.

### 3.3 Residual Networks (ResNet)

CNNs are extensively utilized in computer vision tasks, offering a powerful tool for capturing complex information from visual inputs. Theoretically, a model with an increased number of CNN layers can encapsulate richer information, leading to enhanced performance. However, this model depth enhancement often encounters the degradation problem; as the architecture deepens, the model may begin to lose track of the original input information, which paradoxically results in performance deterioration after a certain number of layers.

To combat this issue, the ResNet [14] framework was introduced, utilizing residual connections to bolster the transfer of original information across each network block. Instead of layers learning a direct mapping H(x), ResNet layers aim to fit a residual function F(x) conceptualized as *F(x) = H(x) – x*. The ResNet layer is thus mathematically articulated as *y = F(x, {W*_*i*_*})+x*.

In instances where F(x) and x have different dimensions, the original ResNet architecture introduces a projection shortcut, resulting in the modified equation *y = F(x, {W*_*i*_*})+W*_*s*_*x*.

We adapted this framework to maintain the original sequence length of the input data x for straightforward interpretation. We achieved this by incorporating padding into the Conv1d layer, thus preserving the sequence length as 42 throughout the computation process.

### 3.4 Multi-head self-attention algorithm

The multi-head self-attention mechanism [31] was used to initiate the processing of the input sequence by deploying separate and learnable linear transformations. These transformations generate queries (Q), keys (K), and values (V), each uniquely parameterized by their respective weight matrices *W*^*Q*^*, W*^*K*^*, and W*^*V*^. Thus, for each attention head indexed by i, the transformed representations are expressed as *Q*_*i*_ *= XW*_*i*_^*Q*^, where *X* denotes the input matrix and *h* is the total number of attention heads.

Attention scores were then computed through the scaled dot product of Q and K, normalized by the square root of the keys’ dimensionality dk, and subsequently passed through a softmax function to derive the attention weights. This is formally articulated as *Attention(Q, K, V) = softmax(QK*^*T*^*/*$\sqrt{\mathrm{dk}}$*)V*.

The multi-head attention framework enables the parallel processing of these attention computations across the multiple heads, where each head yields an output as *Attention(Q*_*i*_*W*_*i*_^*Q*^*, KW*_*i*_^*K*^*, VW*_*i*_^*V*^*)*. The concatenated outputs from all heads, head*i*, are subsequently linearly transformed via the weight matrix W° to produce the final multi-head self-attention layer output, encapsulated by *MultiHead-Attention(Q, K, V) = Concat(head*_*1*_*,…,head*_*h*_*)W*^*O*^.

### 3.5 Focal loss

Focal loss [19] was applied as the loss function to optimize the model, due to the significant imbalance within the dataset, and particularly the disproportionately low number of 5-hydroxymethylcytosine (5hmC) samples compared with other categories of DNA modifications. A prevalence of imbalanced datasets can result in model bias toward the majority class, thus neglecting the minority class, which is undesirable in medical and bioinformatics applications.

Focal loss modifies the cross-entropy loss function by incorporating a modulating factor, which diminishes the contribution of easy-to-classify examples, thereby focusing the model’s attention on the more challenging minority samples. The formula is expressed as *FL(pt) =* - αt * (1 - p_*t*_*)*^*γ*^ * log(p_*t*_*)*.

Here, *p*_*t*_ denotes the predicted probability of the actual class *t*, αt represents the balancing parameter adjusting the importance of different classes, and γ is the focusing parameter that reduces the loss contribution from easy samples. By judiciously selecting *α*_*t*_ and γ, focal loss significantly enhances the model’s ability to recognize minority classes, thereby achieving more equitable and accurate predictions in imbalanced datasets.

### 3.6 Integrated gradient

To elucidate the decision-making processes of complex neural networks, particularly in the realm of text data analysis, the IG technique has emerged as a powerful tool for attributing predictions to input features. This method offers a transparent and mathematically grounded approach for revealing the specific contributions of individual input components to a model’s output. By providing a detailed decomposition of the prediction, IG facilitates a deeper understanding of the model’s behavior, thus enabling researchers and practitioners to pinpoint the exact features that drive the network’s decisions.

Building on this foundation of interpretability, we took a systematic approach to the application of IG in our experiment. First, we identified the neuron corresponding to the input’s label within the output layer, selecting only samples to which the model assigns a probability larger than 0.3. To establish the baseline, we then constructed a data point where all feature values were zero. Commencing from the embedding layer, we calculated the gradients of the input data across the entire model by constructing a smooth interpolation path between the baseline and the actual input, comprising 50 interpolation points. For each point along this path, we computed the gradient of the model’s output relative to the interpolated input, accumulating and averaging these gradients to ascertain the IGs for the entire input dataset. Then, centering on the modification site, segments from each input data and their corresponding IG were extracted, with flanking regions on both the upstream and downstream sides selected as five units in length, resulting in a total flanking region length of 10 units. We used UMAP [21] for sequence embedding and DBSCAN [11] for clustering all extracted segments, and calculated the mean IGs for each cluster. Finally, we constructed the position weight matrix for each cluster to facilitate visualization and used TOMTOM [13] to compare the sequences identified by our model with the motifs discovered by DREME. Segments exhibiting a p-value lower than 0.05 were deemed statistically significant.

### 3.7 Nucleotide masking experiment

In the nucleotide masking experiment, each analytical iteration was confined to a singular motif from an individual organism. The [CLS] token was used for masking purposes, which was conventionally appended at the commencement of all sequences as part of the classification process and thus does not contribute ancillary information to the model’s predictive deliberations. When masking is implemented within the motifs, the modification sites are excluded from being masked, but when masking is executed externally to the motifs, the [CLS] tokens, positioned at the onset of each sequence, are judiciously exempted from the masking procedure. To ensure statistical robustness, each experimental iteration was conducted a minimum of 10 times to ascertain an average metric for analysis.

## 4 Results

### 4.1 Evaluation

A series of parallel investigations were conducted to ascertain the optimal configuration of ResNet and the attention blocks within the network architecture. The marginal utility of the additional attention blocks was observed to diminish beyond a count of two. Similarly, the incorporation of more than five ResNet blocks was found to inversely impact the model’s efficacy. A configuration comprising five ResNet blocks combined with two attention blocks was thus uniformly applied throughout our experimental analysis.

Model performance was rigorously evaluated using a comprehensive set of metrics: accuracy, precision, recall, F1-score, Matthew’s correlation coefficient (MCC), the area under the receiver operating characteristic (ROC) curve, and the area under the precision–recall (PR) curve. As the task at hand is a multi-class classification problem, the evaluation of each class treated instances from all other classes as negative samples. For example, when assessing the performance for 4mC, the negative samples included 5mC, 5hmC, 6 mA, and 6mA-neg, consistent with our classification scheme. In contrast, the overall accuracy of the model was computed as the ratio of correctly classified instances to the total number of instances, as expressed in Equation 5.

Recall = TP / (TP + FN).

Precision = TN / (TN + FP).

ACC = (TP + TN) / (TP + TN + FP + FN).

MCC = (TP * TN – FP * FN) / ((TN + FN) * (TP+FP) * (TN + FP) * (TP + FN)) * * 0.5.

ACC_overall_ = Number of correctly classified instances / Total number of instances.

The experiment was replicated 10 times to ensure reliability, and the mean values were then calculated. The findings together indicate that the model had an accuracy rate of approximately 0.76. The detailed statistics of class- and species-specific performance are given in Table 1, Supplementary Fig. S1, and Table S2.

**Table 1:** Performance summary of iResNetDM.

	Accuracy	Precision	Recall	F1-score	MCC	ROC	RP
4mC	0.887	0.717	0.732	0.722	0.653	0.96	0.84
5hmC	0.964	0.593	0.712	0.644	0.630	0.97	0.72
6 mA	0.884	0.776	0.804	0.788	0.710	0.95	0.87
5mC	0.874	0.692	0.650	0.669	0.592	0.94	0.75
6mA-neg	0.884	0.805	0.781	0.791	0.711	0.95	0.85

### 4.2 A comparison with other deep learning models

To validate the effectiveness of our proposed model architecture, we compared it with two machine learning algorithms—XGBoost and Random Forest—and two other deep learning models: iDNA-ABT [37], which primarily utilizes a self-attention mechanism as its backbone, and Deep4mcPred [38], which integrates ResNet, LSTM, and an attention mechanism (distinct from self-attention). As ours is the first model designed specifically for a multi-class classification task, and the other are all binary classifiers, we modified the output layers of these other models to accommodate multi-class classification while preserving their original architectures. We then trained these models from scratch using the same dataset to ensure a fair comparison.

As shown in Table 2 and Supplementary Table S2, iResNetDM achieves the highest overall performance and demonstrates superior results across all classes. While XGBoost delivers comparable performance to iResNetDM, its effectiveness appears highly dependent on the size of the dataset. For instance, XGBoost excels in datasets like 6 mA and 6mA-neg, where data is more abundant, and achieves the best performance. However, its performance declines significantly with smaller datasets such as 5hmC, where the recall, F1-score, and MCC drop by 0.306, 0.151, and 0.148, respectively, from that of iResNetDM.

**Table 2:** Performance comparison between iResNetDM and other baseline models.

Model	Overall accuracy
Random Forest	0.703
XGBoost	0.742
iDNA-ABT	0.730
Deep4mcPred	0.743
iResNetDM	0.763

iResNetDM also has an advantage in overall accuracy and MCC compared with Deep4mcPred, although Deep4mcPred presents strong competition, particularly in the 5hmC dataset where it performs almost on par with iResNetDM. Nevertheless, iResNetDM consistently outperforms Deep4mcPred in MCC and recall, suggesting that it may be more reliable for certain predictive tasks. Ultimately, iResNetDM offers more consistent and robust performance across diverse datasets.

### 4.3 Ablation experiment

To measure the significance of each component within our model, we conducted a series of parallel experiments with various configurations. The performance of iDNA-ABT [37] shown above underscores the relatively low level of effectiveness of self-attention for this task. Inspired by Zeng and Liao [38], we experimented with incorporating the ResNet architecture into our model. Surprisingly, this significantly improved model accuracy to give a score of 0.763. The addition of each ResNet layer (up to five layers) also markedly enhanced model performance, whereas increasing the number of self-attention layers had a negligible impact. These findings demonstrate that the ResNet architecture is indispensable for enhancing model functionality in predicting DNA modifications.

In addition to evaluating the architectural components of our model, we explored the efficacy of using focal loss, given its pivotal role in our study. The imbalance in our dataset, particularly the markedly lower amount of 5hmC data compared with other categories, initially led to poor precision and recall metrics when using cross-entropy loss, with both consistently below 0.1. However, after implementing focal loss, we observed a significant improvement in these metrics—precision increased to 0.593 and recall to 0.712. These results underscore the effectiveness of focal loss in handling class imbalances by focusing more on hard-to-classify instances, thereby substantially enhancing the predictive capabilities of our model in discerning DNA modifications (Supplementary Fig. S4).

**Fig. 3:**
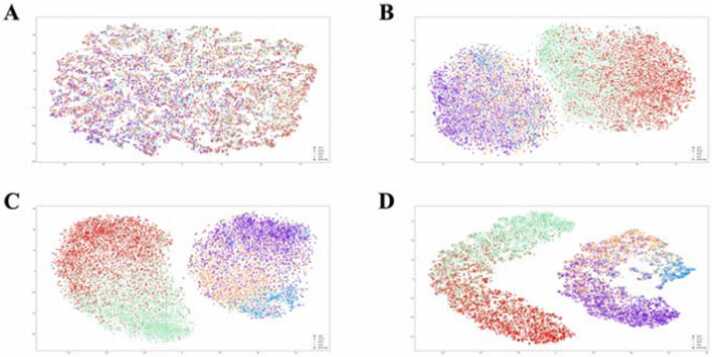
t-SNE visualization of the output extracted from (A): the embedding layer, (B): the ResNet layer, (C): the attention layer, and (D): the output layer.

To validate the functionality and efficiency of each component within our model, we extracted the outputs from each section and used t-SNE [29] for dimensionality reduction and visualization (Fig. 3). The sequential analysis of outputs from various layers of the neural network delineates the integrated functionality and complementary nature of the ResNet and self-attention mechanisms in processing complex features.

**Fig. 4:**
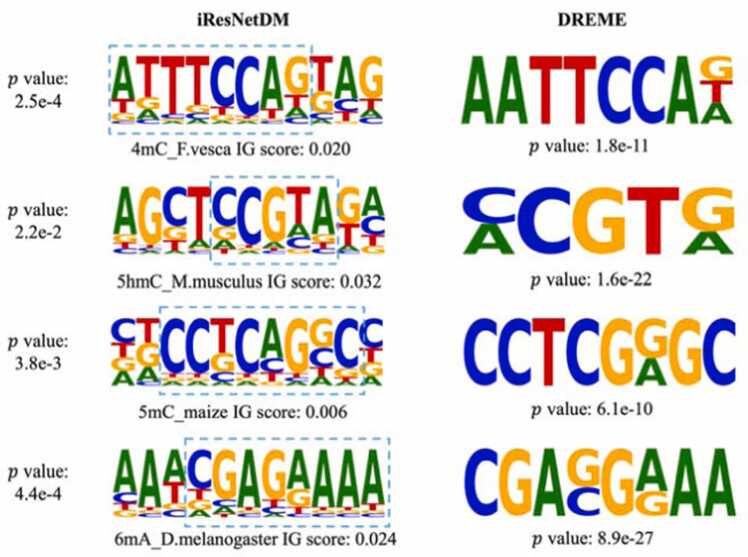
Motif alignment. TOMTOM calculates p-values by comparing observed motifs against a null model composed of motifs from a target dataset, while DREME uses a one-sided Fisher’s exact test to assess the overrepresentation of motifs within a specific dataset.

**Fig. 5:**
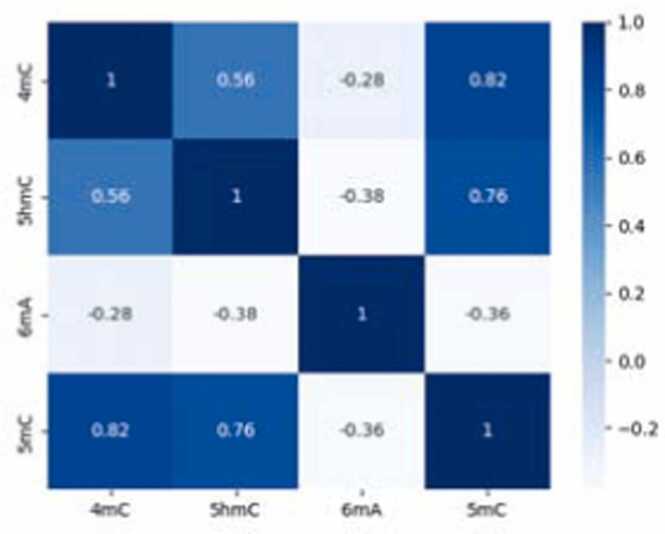
Correlation between different modifications revealed by iResNetDM.

The embedding layer, serving as the initial feature representation, exhibits a dispersed clustering of DNA modifications, thus setting a baseline for subsequent feature enhancement. This layer highlights the raw state of the data, indicating the necessity for substantial transformations to achieve meaningful discriminability. As the first step in this transformation process, the ResNet layers initiate a basic yet crucial separation of features. Although this separation marks an improvement over the embedding layer, it remains insufficient for high discriminability, reflecting the intrinsic complexity of the data involved. The ResNet layers thus establish a fundamental framework for feature differentiation but do not fully capitalize on the potential for fine-grained classification. Building on this framework, the attention layers significantly refine the feature representations. Comparing Fig. 5 (B) and (C) reveals that the attention layers are particularly crucial and effective for the classification of 5hmC modification. The apex of this process is observed in the output from the last hidden layer, where the integration of diverse features results in distinct and refined clustering. These observations highlight the significance of each module in the model, demonstrating that every component is indispensable to the model’s overall capability.

### 4.4 Interpretation

The IG method [26] backtracks from the output to compute the gradient across the model’s architecture. A heightened gradient value signals a more substantial contribution of the corresponding input to the model’s outcome. In our investigation, we applied the IG approach to discern potential motifs. To validate our discoveries, we used an established motif detection algorithm, DREME [3], as a benchmark. The motif comparison tool TOMTOM [13] was utilized to calculate the p-value between the motifs identified by iResNetDM and those found by DREME. Remarkably, the motifs identified by our model aligned closely with those determined by DREME, demonstrating our model’s capability to recognize motifs as crucial for accurate classification.

Further, the investigation revealed distinct motifs in different species, both in terms of sequence and position (Fig. 4 and Supplementary Fig. S2). This finding demonstrates that our model can effectively discern various motifs within the same type of DNA modification, thereby highlighting its robustness and reliability in detecting intricate epigenetic patterns.

To reveal the significance of the identified motifs, we conducted an experimental analysis involving random nucleotide masking, both within and external to the detected motifs, subsequently measuring the impact on the model’s recall score. The outcomes unequivocally suggest that nucleotide masking within the motifs exerts a greater influence on the model’s performance, thereby further confirming the pivotal role of these motifs (Table 3).

**Table 3:** Performance under different configurations.

Model	Accuracy
5 ResNet + 2 self-attention (iResNetDM)	0.763
1 ResNet + 2 self-attention	0.510
3 ResNet + 2 self-attention	0.757
5 ResNet + 5 self-attention	0.763

**Table 4:** Impact of Nucleotide Masking on Model Recall Scores by Motif and Species. The nucleotides marked in red represent the modification sites.

modification	species	motif	mask_inside (std)	mask_outside (std)
4mC	*F. vesca*	ATTTCCAG	0.694 (0.065)	0.706 (0.040)
5hmC	*M. musculus*	CCGTA	0.229 (0.031)	0.403 (0.050)
5mC	*Z. mays*	CCTCAGGC	0.691 (0.049)	0.787 (0.066)
6 mA	*D. melanogaster*	CGAGAAAA	0.891 (0.031)	0.920 (0.028)

The interrelationships among different species have been systematically explored in the research, and intricate patterns of genomic interactions have been revealed [16]. We built on these foundational insights in our study and used an integrated model to further elucidate the associations between various DMM types. For each sequence in our dataset, we carefully extracted the output from the last hidden layer to compute the average representation for each class of DMM.

The correlation heatmap presented in Fig. 5 reveals a consistent negative correlation between 6 mA and other DNA modifications, suggesting an antagonistic regulatory mechanism within the genomic landscape. Conversely, modifications occurring on cytosine—namely 5mC, 5hmC, and 4mC—demonstrated strong mutual correlations. This observation is indicative of co-occurrence and possibly cooperative interactions in specific genomic regions that are extensively modified by multiple types of DMM.

To substantiate this hypothesis, we extended our analysis to the examination of motifs identified by the DREME algorithm (Supplementary Fig. S3). We revealed several motifs that are common across 5mC, 5hmC, and 4mC, thus suggesting their potential significance in epigenetic regulation. These motifs, which are prevalent among highly correlated modifications, may serve as crucial sites for the concerted orchestration of epigenetic marks that modulate gene expression. Our findings suggest that these regions may play pivotal roles in cellular processes, thus warranting further investigation into their biological implications and their overarching impact on gene regulation dynamics.

## 5 Discussion

The current models in DMM research are primarily built on binary classification tasks. While these models are adept at distinguishing the presence or absence of DMMs, they fall short of analyzing the intricate relationships among different types of modifications, thus limiting the scope for more comprehensive analyses. To address this limitation, we introduced the iResNetDM model. This is not only capable of distinguishing between various classes of DMM but also leverages its integrated features to perform a holistic analysis of the relationships among these modifications. The utilization of IG techniques also enhances the model’s interpretability, revealing the motifs that has been intensively modified through various modifications.

In this study, we devised an innovative model that demonstrates exceptional efficacy in discerning four prevalent DMMs. This further confirms the utility of the ResNet architecture in predicting DNA modifications and marks a novel application of ResNet in this domain. The conventional self-attention mechanism, which has shown promise in other contexts, was found to be inferior to the iResNetDM model, which successfully identified several motifs with impressive accuracy across various organisms, thereby highlighting its robustness and broad applicability.

This work pioneers the use of IG techniques in discerning potential motifs associated with DMM, and is thus aligned with other research endeavors [25]. However, compared with the findings of previous studies, we observed improvements in both the breadth and the quality of the identified motifs. For instance, some research [16] lacks the expansive utilization of data that characterizes our approach, potentially limiting its statistical robustness.

Our analysis revealed a distinct pattern of interaction among modification types. The consistent negative correlation between 6 mA and other DNA modifications suggests an antagonistic regulatory mechanism, while strong mutual correlations among cytosine-based methylation modifications (5mC, 5hmC, and 4mC) indicate potential cooperative interactions within certain genomic regions. We further analyzed these regions, which were extensively modified by multiple types of DMM, through the DREME algorithm to identify common motifs. The identified motifs illustrate the potential significance of these modifications in epigenetic regulation, suggesting that they may serve as crucial sites for orchestrating epigenetic marks that modulate gene expression.

Such findings not only underscore the utility of IG techniques in enhancing our understanding of DMM but also suggest that these regions have important roles in cellular processes, thus warranting further investigation into their biological implications and their impact on gene regulation dynamics.

Despite the progress made, constraints regarding the available datasets limited the scope of motif identification for other DNA modifications, such as 6 mA, while some, like 5fC and 5CaC [4], were omitted, due to the absence of corresponding datasets. We anticipate that our model can be applied to novel datasets related to diverse DNA modifications, particularly those associated with adenine such as 6hmA [34], to further substantiate its performance.

## 6 Conclusion

In this work, we have introduced iResNetDM, a deep learning model tailored to predict and distinguish between four specific types of DNA modifications. Our results underscore the capability of iResNetDM to deliver high-performance metrics, demonstrating robust adaptability and efficiency across various DNA modifications and species. This study represents a significant step forward in the field of bioinformatics, particularly in the understanding and prediction of complex epigenetic modifications.

Key contributions of this research include the development of a model that can handle multi-class DMM predictions—a first in the field. By integrating ResNet and self-attention mechanisms, iResNetDM has shown not only to enhance prediction accuracy but also to provide deeper insights into the decision-making processes underlying DMM. This was achieved through the innovative application of the IG technique, which facilitated a clear understanding of the contributions of individual sequence components to the predictions made by our model.

## Data Availability

The datasets and code of our work will be released on GitHub at https://github.com/yaoge777/iResNetDM following publication. For the convenience of future studies, the curated dataset will also be released on GitHub.
