# Supplementary material for: iResNetDM: An interpretable deep learning approach for four types of DNA methylation modification prediction
Source: Comput Struct Biotechnol J. 2024 Nov 13;23:4214–21. doi: 10.1016/j.csbj.2024.11.006 (PMC11621598; doi:10.1016/j.csbj.2024.11.006)
Supplement: Supplementary file 1 — Supplementary material [file mmc1.docx]

| Modification | Organism | Training set | Test set |
| --- | --- | --- | --- |
| 4mC | *C. equisetifolia* | 319 | 46 |
| 4mC | *F. vesca* | 8750 | 1250 |
| 4mC | *S. cerevisiae* | 1723 | 247 |
| 4mC | *Tolypocladium* | 8750 | 1250 |
| 4mC-total |  | 19542 | 2793 |
| 5hmC | *H. sapiens* | 2053 | 291 |
| 5hmC | *M. musculus* | 3191 | 456 |
| 5hmC-total |  | 5244 | 747 |
| 5mC | *Z. mays* | 8750 | 1250 |
| 5mC | NIP | 8750 | 1250 |
| 5mC-total |  | 17500 | 2500 |
| 6mA | *A. thaliana* | 2625 | 375 |
| 6mA | *C. elegans* | 2625 | 375 |
| 6mA | *C. equisetifolia* | 2625 | 375 |
| 6mA | *D. melanogaster* | 2625 | 375 |
| 6mA | *F. vesca* | 2446 | 350 |
| 6mA | *H. sapiens* | 2625 | 375 |
| 6mA | *R. chinensis* | 512 | 375 |
| 6mA | *S. cerevisiae* | 2625 | 375 |
| 6mA | *T. thermophile* | 2625 | 375 |
| 6mA | *Tolypocladium* | 2625 | 375 |
| 6mA | *Xoc BLS256* | 2625 | 375 |
| 6mA-total |  | 26808 | 4100 |
| 6mA-neg | *A. thaliana* | 2625 | 375 |
| 6mA-neg | *C. elegans* | 2625 | 375 |
| 6mA-neg | *C. equisetifolia* | 2625 | 375 |
| 6mA-neg | *D. melanogaster* | 2625 | 375 |
| 6mA-neg | *F. vesca* | 2446 | 350 |
| 6mA-neg | *H. sapiens* | 2625 | 375 |
| 6mA-neg | *R. chinensis* | 512 | 375 |
| 6mA-neg | *S. cerevisiae* | 2625 | 375 |
| 6mA-neg | *T. thermophile* | 2625 | 375 |
| 6mA-neg | *Tolypocladium* | 2625 | 375 |
| 6mA-neg | *Xoc BLS256* | 2625 | 375 |
| 6mA-neg-total |  | 26808 | 4100 |
| Total |  | 95902 | 14240 |

Table S1: Statistic of datasets

| Modification | Organism | Recall |
| --- | --- | --- |
| 4mC | *C. equisetifolia* | 0.609 |
| 4mC | *F. vesca* | 0.859 |
| 4mC | *S. cerevisiae* | 0.765 |
| 4mC | *Tolypocladium* | 0.718 |
| 5hmC | *H. sapiens* | 0.732 |
| 5hmC | *M. musculus* | 0.706 |
| 5mC | *Z. mays* | 0.711 |
| 5mC | NIP | 0.643 |
| 6mA | *A. thaliana* | 0.867 |
| 6mA | *C. elegans* | 0.864 |
| 6mA | *C. equisetifolia* | 0.656 |
| 6mA | *D. melanogaster* | 0.944 |
| 6mA | *F. vesca* | 0.966 |
| 6mA | *H. sapiens* | 0.885 |
| 6mA | *R. chinensis* | 0.851 |
| 6mA | *S. cerevisiae* | 0.811 |
| 6mA | *T. thermophile* | 0.632 |
| 6mA | *Tolypocladium* | 0.776 |
| 6mA | *Xoc BLS256* | 0.805 |

Table S2: Species-specific performance

| Modification: 4mC | | | | | |
| --- | --- | --- | --- | --- | --- |
|  | Accuracy | Precision | Recall | F1-score | MCC |
| Random Forest | 0.873 | 0.669 | **0.760** | 0.712 | 0.634 |
| XGBoost | 0.884 | **0.719** | 0.750 | **0.734** | **0.664** |
| iDNA-ABT | 0.881 | 0.716 | 0.710 | 0.713 | 0.639 |
| Deep4mcPred | 0.886 | 0.714 | 0.732 | 0.723 | 0.652 |
| iResNetDM | **0.887** | 0.717 | 0.732 | 0.722 | 0.653 |

| Modification: 5hmC | | | | | |
| --- | --- | --- | --- | --- | --- |
|  | Accuracy | Precision | Recall | F1-score | MCC |
| Random Forest | 0.946 | 0.661 | 0.05 | 0.09 | 0.175 |
| XGBoost | 0.953 | 0.625 | 0.406 | 0.493 | 0.482 |
| iDNA-ABT | 0.949 | 0.301 | 0.587 | 0.398 | 0.397 |
| Deep4mcPred | 0.961 | 0.605 | 0.705 | **0.651** | **0.634** |
| iResNetDM | **0.964** | 0.593 | **0.712** | 0.644 | 0.630 |

| Modification: 6mA | | | | | |
| --- | --- | --- | --- | --- | --- |
|  | Accuracy | Precision | Recall | F1-score | MCC |
| Random Forest | 0.868 | 0.767 | 0.759 | 0.763 | 0.671 |
| XGBoost | **0.886** | **0.803** | 0.785 | **0.794** | **0.716** |
| iDNA-ABT | 0.882 | 0.762 | **0.807** | 0.784 | 0.704 |
| Deep4mcPred | 0.882 | 0.797 | 0.784 | 0.791 | 0.708 |
| iResNetDM | 0.884 | 0.776 | 0.804 | 0.788 | 0.710 |

| Modification: 5mC | | | | | |
| --- | --- | --- | --- | --- | --- |
|  | Accuracy | Precision | Recall | F1-score | MCC |
| Random Forest | 0.850 | 0.583 | 0.656 | 0.617 | 0.526 |
| XGBoost | 0.870 | 0.641 | **0.678** | 0.659 | 0.580 |
| iDNA-ABT | 0.863 | **0.699** | 0.615 | 0.654 | 0.572 |
| Deep4mcPred | 0.871 | 0.685 | 0.641 | 0.662 | 0.583 |
| iResNetDM | **0.874** | 0.692 | 0.650 | **0.669** | **0.592** |

| Classification: 6mA-neg | | | | | |
| --- | --- | --- | --- | --- | --- |
|  | Accuracy | Precision | Recall | F1-score | MCC |
| Random Forest | 0.868 | 0.757 | 0.764 | 0.761 | 0.669 |
| XGBoost | **0.886** | 0.786 | **0.804** | **0.795** | **0.717** |
| iDNA-ABT | 0.882 | **0.814** | 0.771 | 0.792 | 0.711 |
| Deep4mcPred | 0.881 | 0.777 | 0.789 | 0.783 | 0.702 |
| iResNetDM | 0.884 | 0.805 | 0.781 | 0.791 | 0.711 |

Table S3: Modification-specific performance comparison


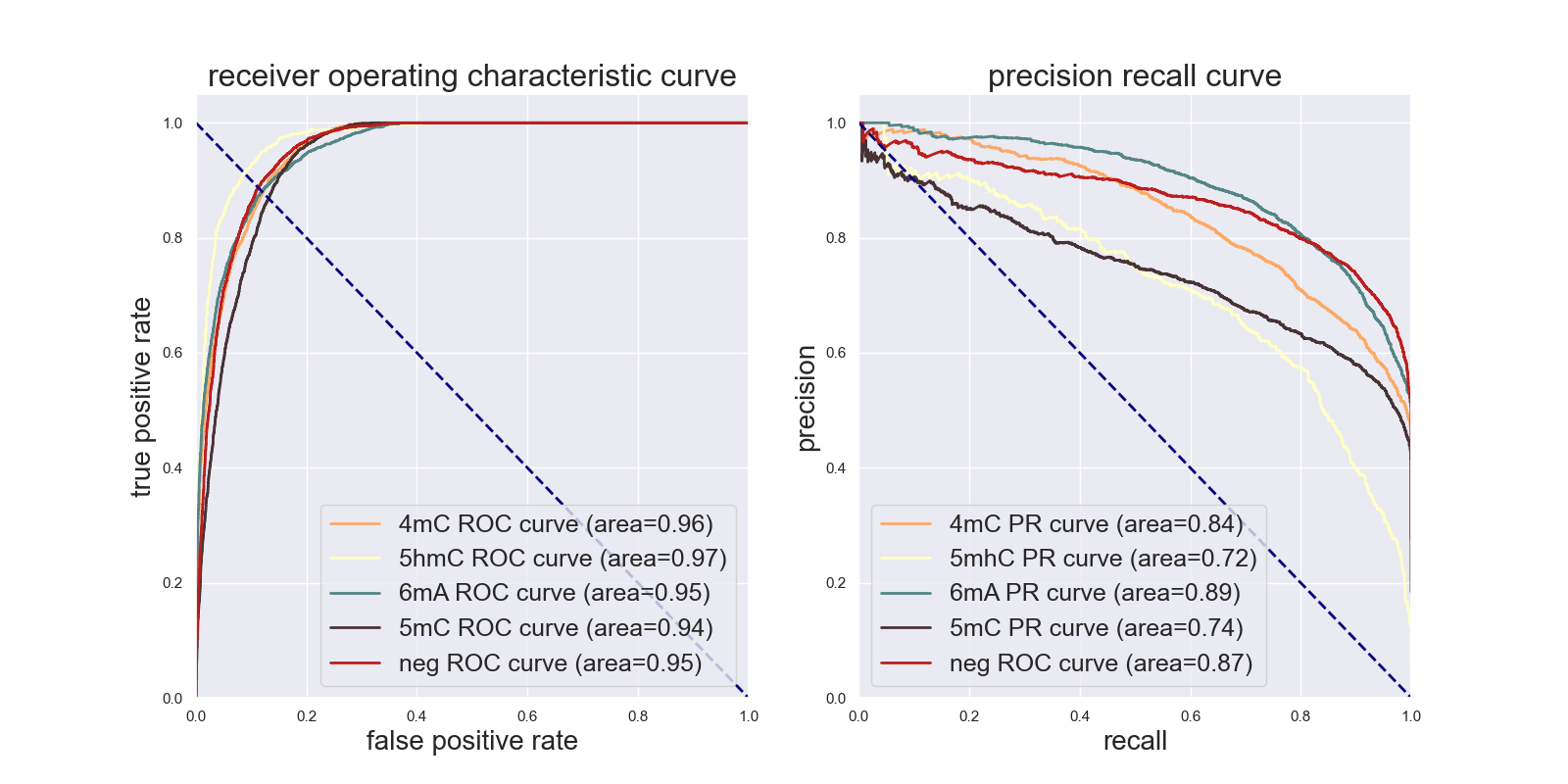


**Figure S1**: Receiver Operating Characteristic (ROC) and Precision-Recall (PR) Curves for the Model


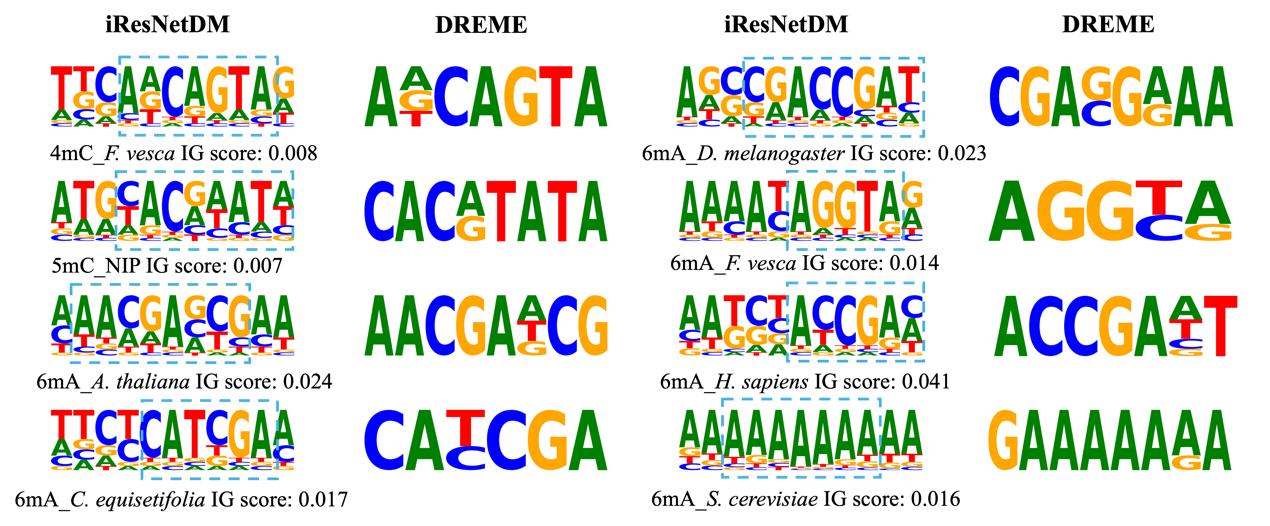


**Figure S2**: Motif alignment. only motifs found by DREME with p-value lower than 0.05 and motif pairs with p-value computed by TOMTOM lower than 0.05 are selected


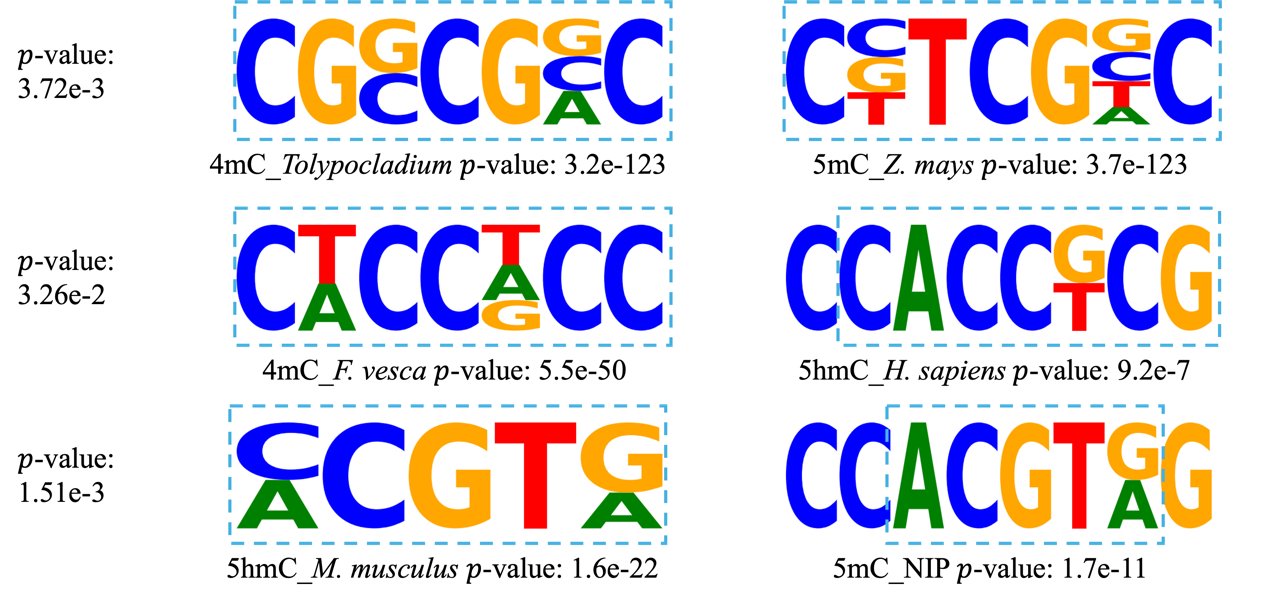


**Figure S3**: Alignment between some motifs of 4mC, 5mC, 5hmC


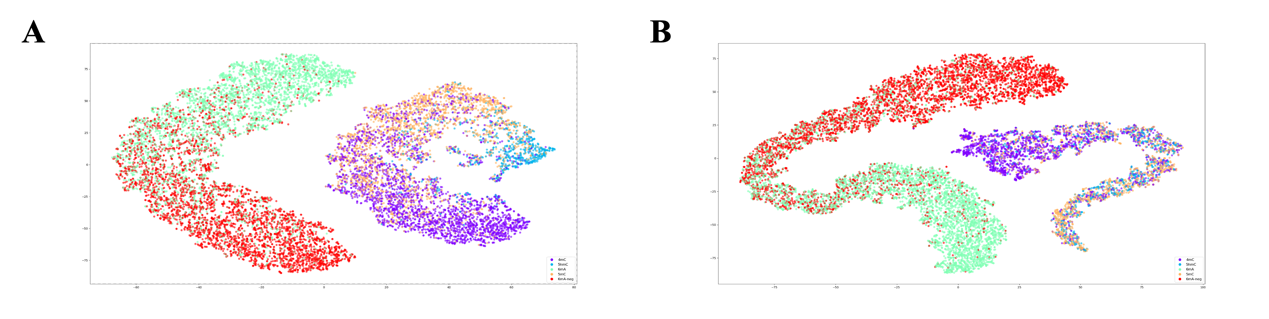


**Figure S4**: t-SNE visualization of the outputs from the model's output layer, comparing the effectiveness of different loss functions: (**A**) focal loss and (**B**) cross-entropy loss. The visualization illustrates that focal loss markedly enhances the model's ability to distinguish 5-hydroxymethylcytosine (5hmC), particularly in cases where sample sizes are limited
